# Catecholamines Differ in Their Capacity to Form Melanin

**DOI:** 10.17912/micropub.biology.001737

**Published:** 2025-09-09

**Authors:** Lin Rayes, David Njus

**Affiliations:** 1 Biological Sciences, Wayne State University, Detroit, Michigan, United States

## Abstract

Upon oxidation, the catechol amino acid L-DOPA polymerizes spontaneously to form the insoluble pigment melanin. Related catechols are less prone to polymerization, however, and this correlates with function. Comparison of the oxidation products of these catechols reveals that L-DOPA, dopamine, norepinephrine and epinephrine form a red-colored “chrome” upon oxidation, but epinephrine and norepinephrine, which act as neurohormones and neurotransmitters, do not go on to form an insoluble melanin. N-acetyldopamine (NADA), which functions in cuticle hardening in insects, does not even cyclize to the “chrome,” so the quinone remains available to crosslink cuticular proteins without obscuring coloration by other pigments. Thus, the side-chains of catecholamines affect reactivity as well as function.

**Figure 1. Oxidation products and melanin formation by different catechols:**
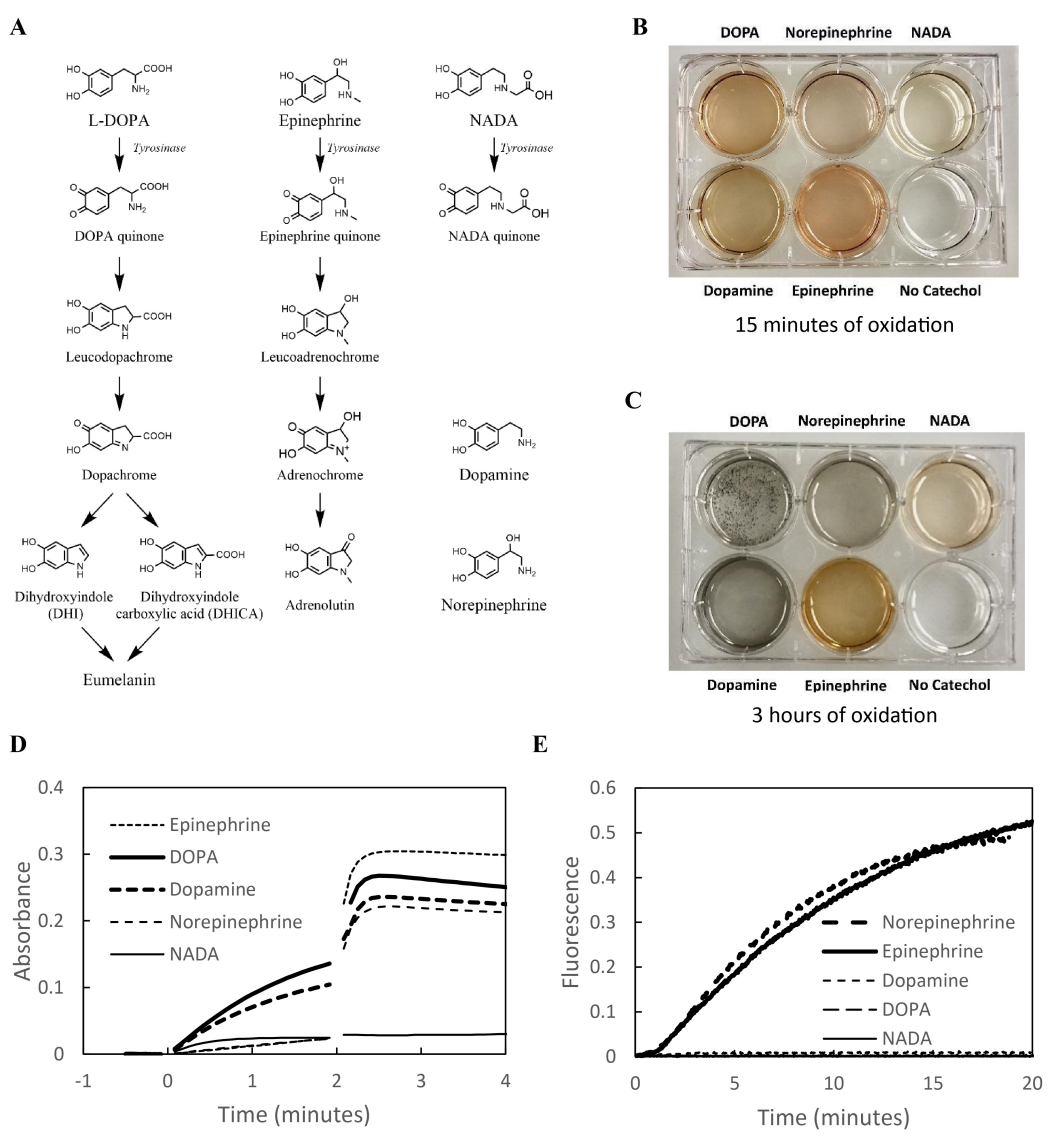
A) Oxidation products of L-DOPA, epinephrine and N-acetyldopamine (NADA). Tyrosinase oxidizes the catechols to the quinone. All subsequent reactions can proceed spontaneously. L-DOPA and epinephrine form a red “chrome” but NADA does not. Dopachrome goes on to yield eumelanin but adrenochrome converts to adrenolutin. B and C) A solution of each catechol (4.4 mM) was oxidized using mushroom tyrosinase at 37°C. B) After 15 min, only NADA fails to show the red color characteristic of a chrome. C) After 3 hours, L-DOPA and dopamine have formed dark insoluble synthetic melanins, while norepinephrine yields little insoluble material and epinephrine and NADA produce none at all. D) Increase in absorbance following oxidation shows formation of “chromes” from all catechols except NADA. Absorbance at 475 nm of a 100 µM solution of each catechol was recorded at room temperature following oxidation by tyrosinase initiated at t=0 and by potassium ferricyanide added at t=2 min. Each line is the average of three replicate traces. E) Formation of fluorescent “lutins” from norepinephrine and epinephrine but not L-DOPA, dopamine or NADA. Fluorescence of a 40 µM solution of each catechol was recorded (excitation = 400 nm; emission = 520 nm) at room temperature. Oxidation was initiated by adding potassium ferricyanide at t=0.

## Description

Catechols, when oxidized to their quinones, are quite reactive and can undergo both internal rearrangement and external additions to yield a variety of products (Schweigert et al., 2001). Of particular importance is the oxidation of L-3,4-dihydroxyphenylalanine (L-DOPA), which results in spontaneous polymerization to create the dark pigment melanin (Cao et al., 2021; d’Ischia et al., 2013; Sugumaran et al., 2020). This polymerization, however, is antithetical to the function of other catechols, such as epinephrine and norepinephrine, which function as important signals in the autonomic nervous system (Wang et al., 2023). An unusual catechol in insects, N-acetyldopamine (NADA), plays a role in hardening regions of the cuticle that are light in color, so reactivity is needed but without pigmentation (Wittkopp et al, 2003; Wittkopp and Beldade, 2009). While enzymatic reactions may contribute to the different fates of these molecules, we show here that there are also inherent differences in the tendencies of these catechols to form melanin. These differences may contribute to their different functions.

In the classic pathway for the biosynthesis of eumelanin, L-DOPA is oxidized to the DOPA quinone (d’Ischia et al., 2013; Sugumaran et al., 2020). This typically requires O _2_ and an enzyme such as tyrosinase (Panel A). Non-enzymatic oxidation by O _2_ also occurs, but much more slowly. The DOPA quinone spontaneously rearranges and oxidizes to form the red-colored dopachrome. This can be seen after 15 minutes of oxidation with tyrosinase at 37°C (Panel B). A red bicyclic chrome is also formed upon oxidation of dopamine, norepinephrine and epinephrine, but not NADA (Panel B). The formation of chromes by L-DOPA, dopamine, norepinephrine and epinephrine can be confirmed by absorbance (Panel D). The fact that NADA does not show an increase in absorbance even after ferricyanide addition rules out the possibility that its failure to form a chrome is because it is a poor substrate of tyrosinase. Apparently, NADA can oxidize to the quinone, but the N-acetyl group prevents cyclization to the chrome.

Dopachrome rearranges to dihydroxyindole (DHI) and dihydroxyindole carboxylic acid (DHICA) (d’Ischia et al., 2013; Sugumaran et al., 2020), which oxidize to indole quinones, that are quite reactive and couple together to form the amorphous polymer that is eumelanin (Panel A). This can be seen after 3 hours of incubation with tyrosinase at 37°C (Panel C). Dopamine also forms a corresponding insoluble melanin. Norepinephrine, however, does so only to a lesser extent, and epinephrine and NADA do not form melanin at all (Panel C). These differences are not explained by substrate specificity of tyrosinase, because, at these catechol concentrations (4.4 mM), oxidation is limited as much by dissolved O _2_ as by tyrosinase activity. Furthermore, the same pattern is observed when the catechols are oxidized with ferricyanide or by autooxidation with O _2_ , although the time scales are different. Ferricyanide converts L-DOPA to melanin in minutes; autooxidation by O _2_ requires days. This confirms that the failure of epinephrine and norepinephrine to form melanin is not because they are poor substrates of tyrosinase; they are simply not as prone to polymerization.

Norepinephrine and epinephrine differ from the other catechols in having a hydroxyl group on the ethylamine side chain (Panel A). This allows them to form the ketone compounds, noradrenolutin and adrenolutin respectively (Palumbo et al., 1989). The fluorescence of these lutins may be observed following oxidation by ferricyanide (Panel E). Note that, while ferricyanide oxidation produces the chrome absorbance immediately (Panel D), there is a delay before the appearance of the lutin fluorescence. This is consistent with the slower conversion of the chrome to the lutin. Also, L-DOPA, dopamine and NADA do not form a fluorescent lutin consistent with their lack of the side-chain hydroxyl group (Panel E). The formation of this lutin is suggestive that norepinephrine and epinephrine have alternative decomposition pathways that do not lead to melanin. Epinephrine is most resistant to melanization, and it is unique in having an N-methyl group. It has been suggested that a methide intermediate is involved in L-DOPA polymerization (Sugumaran and Barek, 2016; Mostert, 2021), and the N-methylation of epinephrine may interfere with formation of this intermediate.

In all animals, the pigment melanin is made by polymerization of L-DOPA (d’Ischia et al., 2013). In insects, dopamine may also polymerize to provide pigmentation and sclerotization or hardening of the cuticle (Wittkopp et al., 2003). Dopamine is also used as a neurotransmitter in both insects and vertebrates, but, in this role, it is confined to the central nervous system in both groups and protected by the blood-brain barrier (Cichewicz et al., 2017). Vertebrates, of course, use epinephrine and norepinephrine as transmitters in the periphery in the autonomic nervous system (Wang et al., 2023). Perhaps their resistance to polymerization is a selective advantage for this peripheral function.

In insects, NADA is used for hardening of the cuticle or sclerotization in regions of the exoskeleton lacking dark pigmentation (Wittkopp et al., 2003; Wittkopp and Beldade, 2009). This happens because the NADA quinone reacts with histidine residues in proteins, thereby cross-linking the proteins (Kramer et al., 2001) and strengthening the cuticle. Consistent with this function, cuticular proteins are rich in histidine in areas where NADA is used (Liu et al., 2021). In this case, it is advantageous that the NADA quinone does not cyclize to the chrome but remains available to react with cuticular proteins. Furthermore, the absence of melanin pigmentation allows for coloration by other pigments or no color at all. This is necessary to form the often spectacular color patterns that insects use for camouflage, mimicry, aposematism and sexual selection (Popadic and Tsitlakidou, 2021).

It should be noted that the focus here has been on melanins formed from pure catecholamines, eumelanin in the case of L-DOPA. Other melanins in animals include pheomelanin and neuromelanin. Pheomelanin incorporates benzothiazines formed from the cysteinyl adduct of L-DOPA in particular. This pigment is lighter in color than eumelanin and its formation depends upon the availability of cysteine (Galvan et al., 2012). Neuromelanin is formed in dopamine and norepinephrine-rich regions of the brain following autooxidation of the catecholamines. Like pheomelanin, it also incorporates benzothiazines derived from the cysteine adducts of these catecholamines. Neuromelanin in the dopamine-rich substantia nigra may be especially relevant to Parkinson’s disease where it may function as a sink for toxic products of dopamine oxidation, and its quantity and composition may also provide a diagnostic indicator of neurodegeneration (Zucca et al., 2023; Cassidy et al., 2019; Cai et al., 2023).

In this connection, it is significant that epinephrine, unlike L-DOPA and dopamine, does not seem to form cysteine adducts that are incorporated into natural melanins. A distinct feature of epinephrine is that the rate at which the quinone cyclizes to adrenochrome is very fast, about 20 times faster than the corresponding rate for L-DOPA quinone and even greater compared to dopamine quinone (Ciolkowski et al., 1992; Land et al. 2003). That means the epinephrine quinone has a much shorter lifetime and will have a correspondingly lower probability of forming the cysteine adducts that are incorporated into pheo- and neuro-melanin. Indeed, Vercruysse (2024) observed that, not only does epinephrine not produce melanin in the absence of cysteine, but pigment formation in the presence of cysteine requires a relatively high concentration of the latter. Thus, not only is epinephrine resistant to forming eumelanin, but it is less prone to forming pheo- and neuro- melanin as well.

In conclusion, polymerization to melanin is the purpose of some catechols (L-DOPA and dopamine) but this property is not an asset for other functions of catechols such as the signaling role of norepinephrine and epinephrine. Moreover, the reactivity of the catechol quinone without pigment formation is necessary for NADA to harden insect cuticles but not darken them. We suggest that evolution has selected catechols with different polymerization properties to fulfill these different roles.

## Methods

To observe the formation of melanin (Panels B and C), each catechol (4.4 mM final concentration) was placed in 2 ml of 50 mM potassium phosphate, pH 6.8 with 25 units of mushroom tyrosinase and incubated at 37°C. Photographs were taken after 15 min (B) or 3 hours (C).

Formation of “chromes” (Panel D) was monitored by recording absorbance at 475 nm using a Shimadzu UV160U spectrophotometer. Oxidation of each catechol was initiated by adding it (100 μM final concentration) to 0.2 M potassium phosphate, 10 μM EDTA, pH 7.4 containing 50 units/ml of tyrosinase. Five microliters of 100 mM K _3_ Fe(CN) _6_ (0.5 mM final concentration) was added after 2 min to oxidize all remaining catechol.

Formation of “lutins” (Panel E) was observed by following fluorescence (400 nm excitation, 520 nm emission) using a Perkin Elmer 204S spectrophotofluorometer. Each catechol was added to 3 ml of 0.2 M potassium phosphate, 1 µM EDTA, pH 7.4 at a final concentration of 40 µM. Oxidation of the catechol was initiated by adding 24 µl of 10 mM potassium ferricyanide (80 µM final concentration).

L-DOPA, dopamine, norepinephrine, epinephrine and mushroom tyrosinase were purchased from Sigma-Aldrich. N-acetyldopamine was obtained from Ambeed, Inc., Buffalo Gove, IL Tyrosinase was dissolved in H _2_ O at a concentration of 5000 units/ml.
